# Phytochemical screening and allelopathic potential of phytoextracts of three invasive grass species

**DOI:** 10.1038/s41598-023-35253-x

**Published:** 2023-05-18

**Authors:** Shaista Jabeen, Muhammad Fraz Ali, Atta Mohi ud Din, Talha Javed, Nouriya Salah Mohammed, Sunbal Khalil Chaudhari, Muhammad Ammar Javed, Baber Ali, Lixin Zhang, Mehdi Rahimi

**Affiliations:** 1 College of Life Sciences, Northwest A&F University, Yangling, 712100 Shaanxi China; 2 Institute of Molecular Biology and Biotechnology, The University of Lahore, Sargodha Campus, Sargodha, 42100 Pakistan; 3 College of Agronomy, Northwest A&F University, Yangling, 712100 Shaanxi China; 4 National Research Center of Intercropping, The Islamia University of Bahawalpur, Bahawalpur, 63100 Pakistan; 5 College of Agriculture, Fujian Agriculture and Forestry University, Fuzhou, 350002 China; 6 Botany Department, Faculty of Science, Sebha University, Sebha, Libya; 7 Institute of Industrial Biotechnology, Government College University, Lahore, 54000 Pakistan; 8 Department of Plant Sciences, Quaid-I-Azam University, Islamabad, 45320 Pakistan; 9 Department of Biotechnology, Institute of Science and High Technology and Environmental Sciences, Graduate University of Advanced Technology, Kerman, Iran

**Keywords:** Plant sciences, Ecology

## Abstract

Undoubtedly, it is important to remain vigilant and manage invasive grasses to prevent their spread and mitigate their negative impact on the environment. However, these aggressive plants can also play a beneficial role in certain contexts. For example, several invasive grasses provide valuable forage for livestock and have disease control potential. Therefore, a research experiment was conducted to explore the pros and cons of this approach, not only for surrounding vegetation but also for human and animal disease control. The study is primarily focused on developing livestock feed, plant-derived herbicides, and an understanding of the phytotoxic effects of invasive species. All plant parts of *Cenchrus ciliaris* L., *Polypogon monspeliansis* L., and *Dicanthium annulatum* (Forssk.) Stapf, were tested for their phyto-chemical screening, proximate, and toxicity analysis which was caused by the methanolic extract of these grass species. Qualitative phytochemical screening tests were performed for proximate composition analysis and toxicity assessment essays. The phytochemical analysis revealed the positive results for alkaloids, flavonoids, coumarins, phenols, saponins, and glycosides, while negative for tannins. Comparison of proximate analysis intimated maximum moisture (10.8%) and crude fat (4.1%) in *P. monspeliensis*, whereas maximum dry matter (84.1%), crude protein (13.95%), crude fiber (11%), and ash (7.2%) in *D. annulatum*. Five (10, 100, 500, 100, 10,000 ppm) and three (10, 1000, 10,000 ppm) different concentrations of methanolic extract prepared from *C. ciliaris*, *P. monspeliansis*, and *D. annulatum* were used respectively for root inhibition and seed germination essay. Furthermore, three different concentrations (10, 30, 50 mg) of plant fine powder were used for sandwich method test. There was a significant decline in the growth rate of experimental model radish seeds (P > 0.005), and results from sandwich method tests showed suppressed growth of root hairs, inhibiting the anchoring of the radish seed. In comparison, results manifest that; *P. monspeliansis* indicated an upsurge of inhibition (66.58% at 10,000 ppm), *D. annulatum* revealed soar germination (75.86% in controlled conditions), and *C. ciliaris* exhibited dramatic shoot up of inhibition because of sandwich method test (14.02% at 50 mg). In conclusion, although grasses are toxic, it is important to consider the beneficiary account.

## Introduction

Due to ever-increasing global population^1^, the quest for maximization of agricultural yield has boosted the use of agricultural inputs to minimize the constraints (nutrient deficiency, and pathogens) in crop production^2^. However, the increased use of synthetic fertilizers, insecticides^3^ and weedicides could deteriorate the agroecosystem that ultimately raises the health concerns for both the humans and animals^4,5^. In this perspective, allelopathic extracts from plants might serve as eco-friendly alternatives for a sustainable agricultural production^6^. For instance, the phytoextracts from allelopathic plants has been recognized as the natural reservoirs of plant growth promoters^7^. In addition, the potential of several phytoextracts as bio-pesticides has also been reported in previous studies^8^. Therefore, the utility of allelochemicals against the pests has attracted the interests of researchers and majority of biopesticides were prepared with an aim to control the insect attack^8,9^. However, the biopesticides for weed management are still very limited.

Invasive crop species pose a complex and persistent challenge for any cropping system, as they aggressively encroach upon and disrupt the growth of primary crops in their vicinity^10^. The ecological and health risks associated with the extensive use of synthetic herbicides, coupled with the scarcity of plant based alternatives have become a pressing issue in modern agriculture^11^. Hence, in today’s agriculture to meet the needs of sustainability, it’s imperative to explore the possibility of using plant-derived materials to combat weeds instead of traditional herbicides^12,13^. Previously, allelopathic extracts from sorghum and traditional medicinal shrubs have shown phytotoxic potential against the weeds in field crop production^14^. Similarly, *Persicaria lapathifolia* inhibit *Echinochloa colona* weed^12^, Artemisia argyi water extract inhibit weeds including *Brassica pekinensis*, *Lactuca sativa*, *Portulaca oleracea*, *Oxalis corniculata*, and *Setaria viridis*^15^. *Ferula assafoetida* L. and *Ricinus communis* L., in concentrations of 0.75% and 1% respectively, limit the germination of *Amaranthus retroflexus* L. weed seeds by around 70%^16^. Taken together, it is suggested that allelochemicals found in certain plants could be explored further to evaluate their natural allelotoxic effects. Previously, the bioassay of lettuce plant was carried out using the sandwich methods and phytoextracts from various invasive herbs and shrubs from different locations of Pakistan and Japan revealed the growth inhibition of lettuce^14^. *P. monspeliensis* has the potential to be used as a decorative, a food source, and considered as significant pasture grass^17^. Similarly, the phytochemical evaluation of naturally occurring and easily available grass species could be very useful in tapping their herbicidal potential as they exist as a persistent and dominant in the agroecosystem.

A consistent finding from previous studies of invasive grasses is that they pose an obvious threat to environment^18^. In contrast, native plants and livestock development were also identified as beneficiaries^19^. Based on proximate composition analysis, targeted grazing of invasive grasses could bring about groundbreaking outcomes, not only for native plants, but also for the climate^20^ and wildlife conservation efforts^21^. Targeted grazing may prove particularly effective in controlling the spread of Buffle grass, an aggressive invader with significant fire hazards^22^. Moreover, qualitative phytochemical testing valued for identification of all chemical constituents which includes presence of protein, carbohydrate, phenol, flavonoids, saponins, and alkaloids was confirmed in extract of different parts of test grass^23^. Plant extracts showed valuable role against pathogenic microbes^24^, all extracts inhibited bacteria which leads to development of inhibition zone^25^. It can then be estimated how grass contributes to different activities, including cattle feed, anti-inflammatory, antimicrobial, antioxidant, and antibacterial properties, management of diseases, drug discovery etc^26^. It is also possible to produce effective drugs for humans and animals based on this phytochemical screening.^27,28^.

Dhaman grass (*Cenchrus ciliaris*), is one of the important grasses in Pakistan with high ethnobotanical uses and all parts of this grass are used in the form of infusion for various purposes^29–31^. It is remarkably resilient and capable of thriving in harsh and constantly fluctuating environment, including high temperature, salinity^32^, heavy metal^33^, intense solar radiation, and minimal moisture^34,35^. This grass disturbs local vegetation and fauna resulting in changes in the available thermal landscape as a result of the invasive plant's disturbance^36^. In addition, the selected grass species are highly competent for CO_2_ assembly as well as they consume nitrogen (N) from the atmosphere and play a vital role in the recycling of N from land^37,38^. It has been utilized widely for medicinal purpose and served as an important feed for grazing animals^39,40^. Previous studies regarded it as a potent grass yielding compounds of high therapeutic values, used by drug-developing companies^41^. The derived compounds from *C. ciliaris* have fungicidal nature and were also effective against bacteria^31,41^, Cyclooxygenase (COX) I and II activity^42^, kidney pain, tumors and wounds, indicating that its phytoextracts are toxic^28,43,44^. *P. monspeliensis* seeds germinate quickly, they could be effective in saline desert soil restoration programmes^45^. It usually develops in the salty ecosystem like sabkhas and can be found from the humid bioclimatic zone to the Sahara. It sustains productivity also in excessive salinity and drought^17^. *P. monspeliensis* (Rabbit's foot grass) plays an essential role in the food, forage, ornamental, and restoration of arid and salt-affected soils because it allows the use of provenances that germinate and establish well under adverse conditions. *P. monspeliensis* has an important role in phytoremediation, especially due to its ability to uptake Ni^46^. Although Polpogan species are recognized for their usefulness as forage and weed plants, their potential as herbicides has not been studied extensively^40,47^. Another grass species, commonly called Forrsk or Marvel grass (*Dichanthium annulatum*) is a perennial and densely tufted grass with rhizomiferous main stems. It is used to treat dysentery and menorrhagia^28^. It is inhabitant of the Middle East, tropical Asia, and parts of Africa. Naturalized in some places, such as Australia^47,48^. *D. annulatum* comprised of Na, Mg, K, Al, Ca, Fe, Si, Sr, Ti, Ba, H, Li, O, N, Ar, and Cs which clarifies its nutritional status. In Pakistan, it has been recorded only from the Punjab, Sargodha, Sheikhupura, and Ladhar. Altogether, these grass species have been utilized as animal feed and medicinal plants.

Keeping in view, the field domination, easy availability of these grasses and their utility in the livestock feed, we designed this study with an aim to screen out the presence of different valuable phytochemicals, to analyze the proximate composition, and to evaluate the dose-dependent toxicity of the phytoextracts from these grasses on the germination and growth rate of indicator species (*Rhaphanus sativus*). The findings of this study may provide a promising direction for developing the potential to livestock feed, plant-derived herbicides, pros and cons underlying the phytotoxic accounts of invasive species.

## Materials and methods

*C. ciliaris*, *P. monspeliensis* and *D. annulatum* were collected from different areas of Punjab. All reagents and chemicals which were used in this experiment and mentioned below were procured from “The University of Lahore”.

### Sample preparation and experimental details

The plant samples of test grasses were collected from Sargodha and Faisalabad regions of Pakistan in April 2018. The Collection and extraction of samples followed the methodology outlined by Arora et al.^41^. The plant were thoroughly washed, dried in the laboratory at room temperature, and then ground into a fine plant powder. The preparation of Methanol extract involved mixing 250 g of plant material with 750 ml of methanol, which was then subjected to soxhlet rotatory apparatus for 36-h. Care was taken to ensure that the temperature did not exceed the boiling point of the corresponding solvent).The Filtrate was separated using Whatman No. 1 filter paper and the resulting extract was placed in beakers and left at room temperature for one week. Afterwards, the extracts were concentrated using a laboratory vacuum rotary evaporator at 40 °C, weighed, labeled, and stored in sterilized bottles at 4 °C for further analysis.

### Phytotoxic effect

The phytotoxic activity of *C. ciliaris*, *P. monspeliensis*, and *D. annulatum* was investigated. Methanol extract of these plants were prepared at Five different concentrations (10, 100, 500, 1000, and 10,000 ppm) and tested for their ability to inhibit root length. In addition, three concentrations (100, 1000, and 10,000 ppm) of the extract were used to assess their effect on germination of radish seeds. A sandwich method using fine powder of all three types of grass (10, 30 and 50 mg) was also employed. The Radish seed germination assay and root inhibition were conducted following Turker and Usta 2008^49^ protocol, while the sandwich method was carried out according to Fuji et al.^50^,

#### Determination of root length inhibition

All seeds were sterilized by using sodium hypochlorite for ten minutes followed by washing with distilled water^51^. Each petri dish (90 mm dia.) lined with two filter paper (Whatman No.1) was positioned in each petri plate and then 5 ml of five different concentrations of extract (10, 100, 500, 1000 and 10,000 ppm) was tipped out in every plate with the help of a pipette. After solvent evaporation, 5 ml distilled water was added to every petri plate. Ten seeds were placed in each petri plate, which was then tightly sealed and incubated at 23 °C. The length of the root of all seeds was measured after 1, 3, and 5 days. Percentage of growth inhibition was measured by the given formula.

$$\mathrm{Growth\,inhibtion\,percentage}=\frac{(100 [\mathrm{PC}-\mathrm{PT}])}{\mathrm{PC}}$$

PT for the length of roots of seeds where extracts were applied (treatment group), and PC for the length of roots where extracts were not used (control group).

#### Determination of germination rate

This experiment was conducted to check the toxic potential of methanolic extract from three different test grasses. Petri dishes with filter papers were prepared in the same way as the root inhibition experiment. However, the key differences were the concentration of extracts and the number of seeds used. In this experiment, hundred seeds of radish were used with three different extract concentrations (100, 1000, and 10,000 ppm). The germination process was observed daily and germination rate was recorded for five days. The germination index was calculated using specific formula.

$${\text{N}} = {\text{Proportion of seeds that germinated on day 1}}$$

where N1, N2, N3

The control group with no treatment was taken as standard. The petri-dishes were kept at room temperature and the germination rate was recorded daily for five days.

#### Sandwich method application for root inhibition rate

In the sandwich method, agar solution (0.5% w/v) was prepared and autoclaved at 121 °C for 15 min. Different amounts of plant material (10, 30, 50 mg) were sandwiched in petri plates. With the help of pipette, 5 ml of agar was applied on the plant material as the first layer, fine powder of *C. ciliaris*, *P. monspeliensis* and *D. annulatum* moved upward, and then powder becomes gelatinous, again plant material placed on this gelatinous powder, and then agar was applied as the second layer. In every Petri plate, five seeds of radish were placed on top agar layer. Petri plates were covered with aluminum foil and placed in an incubator at room temperature. Growth of seeds were observed by measuring the length of the lower plant part (root) and the upper portion (hypocotyl), after 72 h for every growing seed. Effect of extract on root was evaluated using the following equation

$$\mathrm{Growth\,inhibtion\,percentage}=\frac{(100 [\mathrm{PC}-\mathrm{PT}])}{\mathrm{PC}}$$

PT indicates root inhibition from treatment groups and PC showed the control or treatment of 0 concentrations.

### Qualitative phytochemical analysis of plant extracts

Qualitative phytochemical tests were performed according to the protocol of^52^.

#### Test for alkaloids

Mayer’s reagent and Dragendorff’s reagent were prepared to check out the presence or absence of alkaloids. For the preparation of Mayer’s reagent, two solutions were prepared. In one solution, Mercuric chloride (0.356 g) was mixed with 60 ml of water, and for another solution, potassium iodide (5 g) was mixed with 20 ml water. For the preparation of Dragendorff’s reagent, two solutions were prepared, for one solution, 80 ml of distilled water was mixed with 1.7 g of Basic Bismuth nitrate and tartaric acid (20 g), for another solution, 40 ml of distilled water was mixed with 16 g of Potassium iodide. Both prepared solutions were mixed well in 1:1ratio. The mixture of 0.5 g of Plant extract with 8 ml of 1% HCl was prepared, warmed, and filtered. Obtained filtrate was treated to distinguish with both prepared reagents. Turbidity or precipitation is indication of the positive results for test showing alkaloids existence in *C. ciliaris*.

#### Test for flavonoids

To remove fatty material, 0.5 g of freshly prepared extracts were mixed up with petroleum ether. This mixture was joined with 80% ethanol (20 ml) and then filtered. The obtained filtrate was used for mixture preparation of this filtrate and 1% KOH (4 ml). The dark yellow colored filtrate is an indication of positive results for flavonoids.

#### Test for coumarins

0.5 g of extract was taken in a test tube, 1 M NaOH was used to moisten the filter paper, and then the test tube was covered with this filter paper. In a beaker, water was placed on flame for boiling. When the water reaches boiling point, the test tube was positioned in it for a few minutes and then moved out from the water, and paper was removed from the test tube. It was observed in UV light, and yellow fluorescence indicates the presence of coumarins.

#### Test for phenols

FeCl_3_ solution was prepared to identify the phenols. 3–4 drops of this solution were treated with plant extract to identify the phenols. Bluish black color indicates the presence of phenols.

#### Test for saponins

0.5 g of extract was placed in boiling water in a test tube then the tube was cooled at room temperature. Froth formation indicates the presence of saponins.

#### Test for tannins

For the identification of tannins, 0.5 g extract was mixed with distilled water (20 ml) in the test tube, boiled, and this mixture was filtered. The filtrate obtained was mixed with 0.1% freshly prepared FeCl_3_ solution. Brownish-green or bluish-black color is the indication of tannins presence.

### Proximate composition analysis

Previous research shows a connection between natural nutrients and crude extracts utilized in conventional medication^53^. Proximate analysis was carried out to find the percentage of moisture content, dry matter, protein, fat, and crude fiber by following the method^54^. Moisture content was measured according to Nancy Trautmann's method and the same procedure followed by Ashraf et al.^30^. The most famous Kjeldahl method was used to find the crude protein percentage, and fat extraction was carried out with a Soxhlet apparatus. Acid–base treatments were used to estimate the crude fiber percentage.

#### Moisture content percentage

The moisture content of the grass was calculated according to the protocol of Nancy Trautmann and Tom Richard. Firstly, a small container was weighed and then 1 g of plant material was put into placed in the oven at 105–110 °C for duration of 24 h to remove any moisture. After that, the sample was weighed again, and the weight of the container was subtracted. The moisture content was calculated using the following formula.

$$Mn=\frac{(\mathrm{Ww}-\mathrm{Wd})}{(\mathrm{Ww}\times 100)}$$

Mn for moisture content (%) of material n and WW for the wet weight of the sample, and Wd is the weight of the sample after drying.

#### Dry matter percentage

According to AOAC methods described by Poitevin et al.^55^ dry matter was determined by using the formula

$$Dry\,Matter\,percentage = { 1}00 - Moisture\,\,Content$$

#### Protein percentage

The total nitrogen content of the samples was determined by the micro-Kjeldahl method. Finely ground material (1 g) was put in a digestion flask with 3 g of digestion mixture (mercury sulfate (HgSO_4_) and potassium sulphate (K_2_SO_4_) at a ratio of 1:9 and 20 ml concentrated H_2_SO_4_. The samples were boiled in a digestion apparatus for about two hours until the contents became clear. The digested material was diluted to 250 ml. Ten ml were transferred to the micro Kjeldahl distillation apparatus and distilled in the presence of 50 mg Zn dust and 10 ml NaOH (40%). The distillate was collected in a receiver containing 5 ml boric acid (2%) and methyl red as an indicator solution. The contents of the receiver were titrated against sulfuric acid to a light pink color endpoint. From the volume of acid, the percentage of nitrogen was estimated, and protein was determined using the formula:

$$Nitrogen\,Percentage=\frac{(\mathrm{Volume\,of\,}0.1\mathrm{\,N\,H}2\mathrm{SO}4 \times 0.0014 \times 250 \times 100)}{(\mathrm{Weight\,of\,Sample }\times 10)}$$

$${\text{Protein Percentage}}\, = \,{\text{N}}\, \times \,{6}.{25}.$$

#### Fat percentage

A dried sample was extracted with petroleum ether (400C–600C) in a Soxhlet apparatus to estimate the lipid contents to remove the ether soluble component. The extracted material was dried to a constant weight in an oven at 700 °C. The lipid content was calculated using the following formula:

$$\mathrm{Fat\,Percentage}=\frac{(\mathrm{Weight\,of\,Ether\,Extract}\times 100)}{(\mathrm{Weight\,of\,Sample})}$$

The planting sample was boiled in the presence of 1.25% NaOH, followed by 1.25% H_2_SO_4_ to dissolve alkali and acid-soluble components. The residue containing crude fiber was dried to a constant weight. The loss of weight on ignition in a muffle furnace at 500 °C was used to calculate the crude fiber.

#### Ash percentage

A dried (1 g) sample was carbonized on an oxidizing flame until no fumes came out. It was then ignited at 600 °C in a muffle furnace to burn off all organic matter.

$${\text{Ash Percentage }} = {\text{Weight of Ash }} \times { 1}00$$

### Data analysis

The data was sorted by Microsoft Excel 2010 software, statistical analysis was performed using Statistix 8.1 (Analytical Software, Tallahassee, FL, USA). One-way analysis of variance (ANOVA) was performed to detect differences among treatments. The least significant difference (LSD) test was performed to ascertain significant differences between means. A value of p < 0.01 denoted statistical significance.

### Ethics approval and consent to participate

C. ciliaris, P. monspeliensis and D. annulatum were collected from University of Lahore, Lahore, Pakistan. All the experiments were performed in accordance with relevant guidelines and regulations".

## Results

### Toxicity analysis

#### Root length inhibition in radish seeds by methanolic extracts of selected grass species

**Figure 1:**
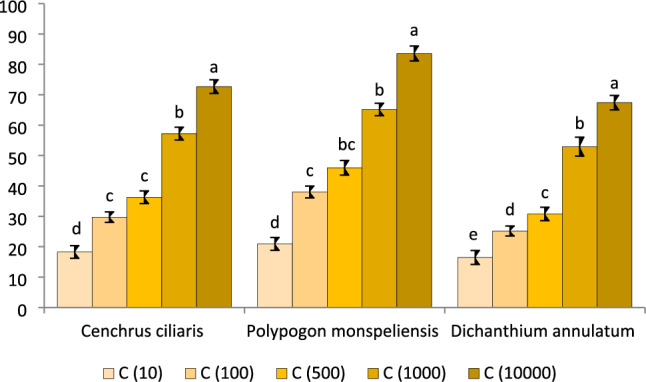
Root inhibition of radish seedlings at different methanolic concentrations of C. ciliaris, P. monspeliensis and D. annulatum methanolic extract.

Radish (*Rhaphanus sativus*) seeds were utilized to evaluate the toxicity levels of the extracts from three grasses. The selection of radish seeds was based on their ease of germination, affordability, rapid growth rate year-round availability. The experiment was carried out under controlled conditions, using methanol extract at a concentration of 10 ppm, and the maximum root length was observed from the first to the fifth day of germination, while root inhibition was at its minimum. Inhibition in root length was increased by increasing the concentration of methanol extracts, with the maximum inhibition observed at 10,000 ppm. Furthermore, when the methanol extract concentration was 10,000 ppm, *P. monspeliensis* showed the highest root inhibition (83.58%), followed by *C. ciliaris* (73.39%) and *D. annulatum* (67.42%) as demostrated in Fig. 1.

#### Germination rate of radish seeds by methanolic extracts of selected grass species

**Figure 2:**
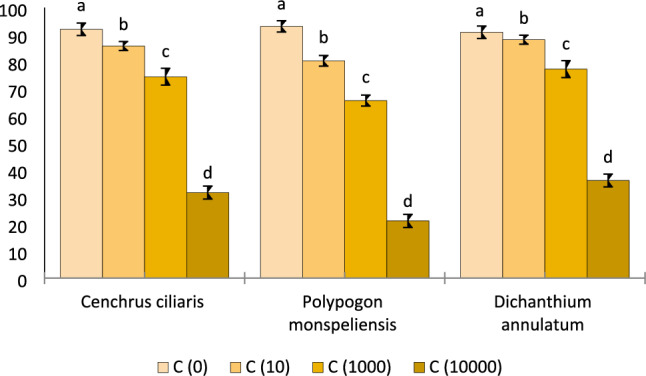
Germination rate of radish seedlings at different concentrations of C. ciliaris, P. monspeliensis and D. annulatum methanolic extract.

**Figure 3:**
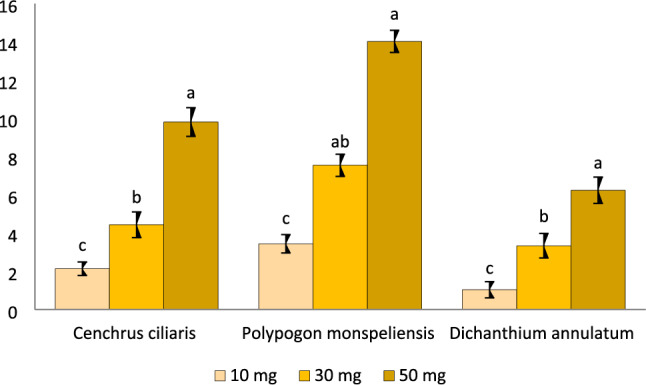
Inhibition rate of both root and hypocotyl of radish seedlings at different amount of C. ciliaris, P. monspeliensis and D. annulatum plant powder.

Data regarding germination rate of radish seeds by methanolic extracts of *P. monspeliensis*, *C. ciliaris* and *D. annulatum* is presented in Fig. 2. The maximum germination rate was observed in controlled conditions (no methanol extract) from the first to the fifth day of germination. The germination rate of seeds was decreased linearly by increasing concentrations of prepared methanol extract. The minimum germination rate was found to be 21.12%, 31.6%, and 36.07% by methanol extract of 10,000 ppm of *P. monspeliensis, C. ciliaris*, and *D. annulatum* respectively, as depicted in Fig. 3.

### Sandwich method

Petri dishes were sterilized and used for three replications of each selected amount (10, 30, 50 mg) of plant material. Two layers of Agar gel were used to sandwich the plant material. and seeds were positioned on the top agar layer. The petri dishes were then covered with aluminium foil and kept in dark for 72 h to observe any inhibition in root length and hypocotyl. The addition of plant powder led to an increase in inhibition rate. The experiment results showed that, in comparison with the control group, the, maximum inhibition rates for *the P. monspeliensis*, *C. ciliaris*, and *D. annulatum* methanolic extractswere 14.02%, 9.80%, and 6.23%, respectively, as illustrated in Fig. 3.

### Qualitative phytochemical analysis of ***C. ciliaris***, ***P. monspeliensis*** and ***D. annulatum***

Various biological activities of plants may be determined by evaluating their chemical ingredients. In the present investigation, qualitative phytochemical analysis was performed by methanol extract of all three kinds of grass. Phytochemical screening showed that alkaloids, flavonoids, phenols, coumarins, and saponins were present in *C. ciliaris*, *P. monspeliensis*, *D. annulatum*, while tannins were absent in all the selected grass species, as shown in Table 1.

**Table 1:** Phytochemical analysis of C. ciliaris, P. monspeliensis and D. annulatum by methanolic extract.

Phytochemicals	C. ciliaris	P. mospeliensis	D. annulatum
Alkaloids	+	+	+
Flavonoids	+	+	+
Phenols	+	+	+
Coumarins	+	+	+
Saponins	+	+	+
Tannins	−	−	−

### Proximate analysis

When considering the significance of flora and determining the health content of plants, it is important to evaluate their chemical composition. In addition to providing feed quality information, proximate analysis can also estimate TDN and energy consumption of animals in different species and classes. It plays a vital role in assessing the suitability of plants species for the requirement of various ruminants. Crude protein and digestible nutrients are two factors associated with feeding value. The production of milk, meat, and reproduction of animals are all dependent on crude protein. C. ciliaris, P. monspeliensis and D. annulatum were analyzed for proximate composition analysis of moisture, crude protein, crude fat, crude fiber, Dry matter, and Ash values have been presented in Table 2.

**Table 2:** Percentage of different contents in C. ciliaris, P. monspeliensis and D. annulatum.

Nature of sample		C. ciliaris	P. mospeliensis	D. annulatum
Dry matter %	71.11		79.2	84.1
Moisture %	5.8		10.8	5.3
Crude protein %	7.25		12.25	13.95
Crude fat %	3.22		4.1	2.11
Fiber %	9		1.4	11
Ash %	3.2		5.5	7.2

## Discussion

Phytochemicals are plant-derived compounds; this group also includes secondary metabolic chemicals^56,57^. Phytochemicals are majorly accountable for the therapeutic role of plants^28^. These compounds increase the nutrient absorption and utilization in animals, boost their immune system, and enhance their overall health and productivity^58^. Our research focused on three invasive grass species and identified the toxicity level, phytochemical analysis and proximate composition. According to phytochemical screenings conducted on selected invasive grasses, alkaloids, flavonoids, phenols, coumarins, and saponins were detected in *C. ciliaris*, *P. monspeliensis*, and *D. annulatum* while tannins were not detected. Alkaloids act as natural insecticides and can protect the animals from insect-borne diseases^59^. Flavonoids and phenols have antioxidant properties and can scavenge harmful free radicals, reducing oxidative stress and inflammation^60–62^. Alkaloid and saponins in the plant make the plant extracts potential antifungal agents^63^. Saponins improve digestion and absorption of nutrients by increasing the permeability of the gut wall, thereby improving feed efficiency^64^. Thus, the presence of these phytochemicals in the test grass suggests that it can enhance the health and performance of livestock. Moreover, absence of tannins in the test grass is an advantage for livestock as tannins interfere with nutrient absorption and reduce food palatability. Tannins bind with protein and carbohydrates and make feed unavailable for digestion. Tannins can cause astringency and bitterness which affect the food acceptance by animals^65^. Overall, the absence of anti-nutritional factors in the test grasses suggest that it can be a good forage option for all livestock.

The phytotoxic results of seed germination, root inhibition and sandwich method experiments suggest that the tested grasses have some level of toxicity that can be harnessed as a natural herbicide. The tested grasses showed phytotoxicity due to presence of certain phytochemicals that can be used as natural herbicide. This offers a promising and eco-friendly solution for farmers seeking alternatives to synthetic herbicides that can harm the environment and human health. The grasses can inhibit weed growth by interfering with their metabolic processes or disrupting their cell membranes. This natural herbicide can be cost-effective, sustainable, and a valuable contribution to sustainable agriculture.

*Polypogon monspenliens* can produce more *phytosiderophores* and organic acids, including citric, acetic, oxalic, and malic acids. It is suitable for pasture grass; it prevents iron chlorosis in calcareous soils and intercropping systems with fruit trees^17^. The bioactive compounds found in *D. annulatum* include flavonoids, terpenoids, alcohols, phenols, and fatty acids. Hexadecanoic acid (20–38%) was found to be more abundant than other compounds that aid in disease prevention^28^. Results demonstrated that methanolic extracts of test invasive grasses impacted the development of sample radish seeds. When compared to root length, the germination process was less suppressed. Roots are sources of mineral absorption and collection, so plant extract significantly impacts roots^66,67^. Turk et al.^68^ explained that developing phenomena are more vulnerable to phytotoxic allelochemicals than germination. Integument seeds were not in direct contact with toxic plant extract; roots were disturbed more than seeds as stunted root growth was observed. Seeds were less affected; it could be because their integuments protected them. Dandelot et al.^69^ noted that sample seeds were less sensitive to phytotoxins than seedlings. Phytoextract toxicity inhibits plant root and aerial development even if seeds germinate^70^. These findings demonstrated that the most significant concentrations of methanol extracts hinder plant development and might be investigated further to develop plant-based herbicides. *P. monspeliensis* has a high biomass production rate. At 100 mm NaCl salinity, seed germination was shown to be drastically reduced (8% germination rate) (P < 0.0001, F = 43.133), but seed germination continues even at 300 mm NaCl. Maximum growth is possible at light salinity (50 mm)^45^.

Sandwich method approach was helpful to find out the toxic nature of the plant. Anjum and colleagues used the sandwich approach to investigate the substantial inhibitory effects of *Albizia lebbeck* and *Broussonetia papyrifera* during examining the inhibitory influence of 14 medicinal plants^71^. Another research utilised the sandwich technique to assess the inhibitory impact of *Ziziphus spina-christi*, Desf., *Juglans regia*, *Lavandula stoechas*, *Artemi-siaherba-alba* Asso., *Rosmarinus officinalis*, and *Cenchrus ciliaris* on chicory (*Cichorium pumilum*) and berseem (*Trifolium alexandrinum*). Seven different amounts (0, 2.5, 3.75, 5.6, 6.5, 7.5, and 12.5%) of plant powder were used and resulted in considerable (51%) inhibitory impact of *C. ciliaris* on berseem seeds at the maximum amount of plant powder^72^.

A relatively small amount of ash was found in our sample when compared to the other chemical ingredients. Several factors that impact ash content include; Weather, drought, humidity, maturity stage, and the sample acquired at a particular season^15^. Plants require various nitrogenous foods for vegetative development. The three fundamental building elements of life are protein, carbs, and fat. Proteins found in seeds are crucial for plant nutrition^73,74^. Plant stores protein well in the early stages of development; it is then used during flowering and fruiting and during the dormant period when their nutritional status deteriorates^75^. Ruminants’ crude protein consumption in the diet varies from 7 to 20%, depending on the species, sex, and physiologic condition^76^. Our test grasses had CP values ranging from 7.25 to 13.95%, indicating suitable for animal feed. The threshold value of roughly 3.6% crude proteins in feed is mandatory^77^. *D. annulatum*, one of our test grasses, had the highest CP level (> 13). CP concentrations greater than 13% indicate that high protein-containing range plants, especially shrubs, can be utilized to supplement poor-quality roughages to boost ruminant livestock^78^. *D. annulatum* have nutritional components (mainly magnesium) in considerable concentrations, which could be exploited in the food and pharmaceutical industries^28^. Lipids are an excellent energy source and help transport fat-soluble vitamins, protect and preserve vital tissues, and conduct essential cell functions. *D. annulatum* consist of several hydrocarbons, fatty acids, alcohols, and volatile chemicals. Plants benefit from moisture content because it regulates food processing, storage, and transportation^79^. Sasoli et al.^77^ reported 28.08% CP, 3.02% EE, and 5.15% ash in *Polupogan monspeliensis*, whereas *Cenchrus ciliaris* had 20.56% CP, 3.10 EE %, and 19.59% ash. The crude fiber in food indicates the presence of non-digestible carbohydrates and lignin. Crude fiber assists food digestion; Its excess may result in intestinal ailment, reduced edibility, and less nutritional use^80,81^. Kirwa et al.^82^ observed significant variations among *C. ciliaris* ecotypes and crude fiber levels ranging from 38.4 to 32.4%. Hoyam and coworkers found similar results; they suggested *Cenchrus ciliaris* composition is suitable for livestock, research results showed 92.17% DM, 91.14% OM, 14.41% CP, 0.87% EE, 55.88% ADF, 75.00% NDF, 7.50% ADL, 11.15% NFE, 10.80% IVOMD, and 1.73% ME in *Cenchrus ciliaris*^83^.

The proximate composition results indicates that the selected grass species are highly effective for livestock. The high dry matter percentage shows high nutrient content per unit weight, which is valuable for meeting the nutritional requirements of livestock^84^. The low moisture content indicates that the grass can be easily stored without the risk of spoilage^85^. High crude protein provides essential amino acids required for growth, maintenance and repair of animal tissues. Crude fat provides a source of energy for the animals, while the fiber content help in digestion and maintaining gut health^86^. The ash content in the grass provides essential minerals for the animal health. Overall, the proximate composition results suggest that the experiment grasses are highly effective and nutritious feed for livestock, which can promote productivity, and overall health.

## Conclusion

The current study assessed the poisonous effects of methanol extracts from all test plants (*C. ciliaris*, *P. monspeliensis*, and *D. annulatum*) through radish seed germination assay, root inhibition assay, and sandwich method. So, this study demonstrated that all three grass species show bioactive toxic principles. Phytochemical tests resulted positive for all except tannins. We concluded that these plants have toxic effects on the growth and germination and could be explored in detail to check their utility in herbicide formation. Besides, it also has many positive impacts for livestock feed and agriculture purpose. However, additional toxicity research is required to know about its quantity alteration and for separation and structural information of these bioactive compounds accountable for the toxicity.

## Data Availability

All data generated or analysed during this study are included in this published article.
